# Mechanisms of endothelium-dependent relaxation evoked by anandamide in isolated human pulmonary arteries

**DOI:** 10.1007/s00210-014-0961-9

**Published:** 2014-02-28

**Authors:** Marta Baranowska-Kuczko, Hanna Kozłowska, Mirosław Kozłowski, Eberhard Schlicker, Monika Kloza, Arkadiusz Surażyński, Emilia Grzęda, Barbara Malinowska

**Affiliations:** 1 Department of Experimental Physiology and Pathophysiology, Medical University of Białystok, Mickiewicz Str. 2A, 15-222 Białystok, Poland; 2 Department of Clinical Pharmacy, Medical University of Białystok, Mickiewicz Str. 2A, 15-222 Białystok, Poland; 3 Department of Pharmacology and Toxicology, University of Bonn, Sigmund-Freud-Str. 25, 53127 Bonn, Germany; 4 Department of Thoracic Surgery, Medical University of Białystok, M.C. Skłodowska Str. 4A, 15-276 Białystok, Poland; 5 Department of Medicinal Chemistry, Medical University of Białystok, Mickiewicz Str. 2D, 15-222 Białystok, Poland

**Keywords:** Human pulmonary artery, Anandamide, Nitric oxide, Prostacyclin, Potassium channels, O-1918-sensitive cannabinoid receptor

## Abstract

Endocannabinoids contract, relax or do not affect vessels with different calibre and tone in the pulmonary circulation in four species. The aim of the present study was to determine the mechanisms involved in the anandamide-induced relaxation of human pulmonary arteries (hPAs). Studies were performed in the isolated hPAs pre-constricted with the prostanoid TP receptor agonist, U-46619. To detect fatty acid amide hydrolase (FAAH) expression, Western blots were used. Anandamide concentration dependently relaxed the endothelium-intact hPAs pre-constricted with U-46619. The anandamide-induced relaxation was virtually abolished by removal of the endothelium and strongly attenuated by inhibitors of cyclooxygenases (indomethacin, COX-1/COX-2, and nimesulide, COX-2), nitric oxide synthase (*N* ^*G*^-nitro-l-arginine methyl ester) given separately or in combination, FAAH (URB597), and the prostanoid IP receptor antagonist, RO1138452. The anandamide-evoked relaxation in the endothelium-intact vessels was attenuated in KCl pre-constricted preparations or by the inhibitor of large-conductance Ca^2+^-activated K^+^ channels, iberiotoxin. In experiments performed in the presence of URB597 to exclude effects of anandamide metabolites, the antagonist of the endothelial cannabinoid receptor, O-1918, diminished the anandamide-evoked relaxation whereas the antagonists of cannabinoid CB_1_, CB_2_ and vanilloid TRPV1 receptors, AM251, SR144528 and capsazepine, respectively, had no effect. Western blot studies revealed the occurrence of FAAH protein in the hPAs. The present study shows that anandamide breakdown products, cyclooxygenase pathways, nitric oxide, potassium channels and the O-1918-sensitive cannabinoid receptor play a role in the anandamide-induced relaxation of the hPAs with intact endothelium.

## Introduction

Pulmonary arterial hypertension (PAH) is a progressive, potentially lethal disease. Patients with PAH have increased levels of potent vasoconstrictors and decreased levels of the endothelial vasodilator nitric oxide (NO) and prostacyclin (PGI_2_), leading to enhanced vasoconstriction, thrombosis, and pulmonary vascular remodelling (Frumkin 2012). Available therapies for PAH target the imbalance of vasoconstricting and vasodilating mediators leading to vasorelaxation in pulmonary vasculature (Frumkin 2012; Waxman and Zamanian 2013; Benyahia et al. 2013). However, to date, PAH is incurable with the currently approved medications, and there is an urgent need for alternative treatment strategies. Hornig (2007) has suggested that endocannabinoids may represent a future option in the PAH therapy.

The two best known endocannabinoids, anandamide (*N*-arachidonoylethanolamine) and 2-arachidonoylglycerol (2-AG), influence the diameter of systemic blood vessels by various mechanisms (Montecucco and Di Marzo 2012), and their possible role has also been studied in the pulmonary vasculature. Endogenous anandamide was detected in the lung of the rabbit (Wahn et al. 2005), the mouse (Wenzel et al. 2013) and the rat (Calignano et al. 2000), and endogenous 2-AG was found in the rabbit lung (Wahn et al. 2005). Moreover, fatty acid amide hydrolase (FAAH), the enzyme responsible for anandamide degradation, was detected in rabbit and murine lungs (Wahn et al. 2005; Wenzel et al. 2013) and in human pulmonary artery smooth muscle cells (Meng et al. 2008; Wenzel et al. 2013). However, only few functional studies concerning direct actions of endocannabinoids on the pulmonary vasculature have been published in the latter four mammalian species in vivo or in vitro.

In the isolated, ventilated, and buffer-perfused lung of the rabbit, both anandamide and 2-AG increased the pulmonary arterial pressure whereas *R*-methanandamide (a metabolically stable analogue of anandamide) and noladin ether, a third endocannabinoid (which is also metabolically stable), failed to do so (Wahn et al. 2005). These data are similar, although not identical, to those obtained in the isolated perfused lung system of mice where anandamide but not 2-AG or *R*-methanandamide increased the pulmonary arterial tone (Wenzel et al. 2013). The explanation for the vasopressor effect of anandamide is that this endocannabinoid is degraded by FAAH into arachidonic acid products which in turn are converted to vasoactive agents by cyclooxygenase-2 (COX-2) (Wahn et al. 2005) and COX and/or lipooxygenase (Wenzel et al. 2013). The involvement of cannabinoid CB_1_ receptors could be discarded in the rabbit and mouse lungs (Wahn et al. 2005; Wenzel et al. 2013). With respect to the rabbit lung, the vanilloid transient receptor potential cation channel of V1 type (TRPV1) receptor could also be excluded (Wahn et al. 2005), which, like CB_1_ or CB_2_ receptors, is a typical target of anandamide and is known to elicit dilatation in some vascular beds of the systemic circulation (for review, see Montecucco and Di Marzo 2012). On the other hand, in another preparation of rabbits, i.e. pre-constricted, isolated pulmonary arteries, noladin ether relaxed the rabbit pulmonary artery smooth muscle (Su and Vo 2007). Two mechanisms are involved in the latter effect, namely, the activation of cannabinoid CB_1_ receptors and, in addition, the activation of O-1918-sensitive receptors. The molecular and biological properties of the latter receptors that have been identified in a series of vascular beds of the systemic circulation (Montecucco and Di Marzo 2012) are so far poorly understood.

The effect of endocannabinoids has also been studied in isolated pulmonary arteries of mice, rats and humans. In non-pre-constricted preparations of rats and mice, anandamide was devoid of an effect (Baranowska-Kuczko et al. 2012; Wenzel et al. 2013). On the other hand, anandamide induced a vasorelaxant effect in pre-constricted pulmonary arteries from rats (Baranowska-Kuczko et al. 2012) and humans (Kozłowska et al. 2007). In rat pulmonary arteries, this effect involves O-1918-sensitive but not CB_1_ and TRPV1 receptors; in addition, COX products play an important role (Baranowska-Kuczko et al. 2012). With respect to human pulmonary arteries (hPAs), virodhamine (a fourth endocannabinoid) shows a vasorelaxant effect again related to the activation of O-1918-sensitive receptors and the formation of COX products (Kozłowska et al. 2008). Our previous data (Kozłowska et al. 2008) do not allow to conclude whether CB_1_ and TRPV1 receptors play a role as well since virodhamine has a low or missing intrinsic activity at CB_1_ receptors and does not bind to TRPV1 receptors (for review, see Pertwee et al. 2010). Moreover, anandamide, which showed a vasodilatory effect as well, has been studied in the presence of a CB_1_ receptor antagonist only, and the mechanisms of its vascular effect have not been examined (Kozłowska et al. 2007). So, the aim of the present study was to examine the receptors and mechanisms involved in the effect of anandamide in hPAs. We found that the anandamide-induced vasorelaxation is strongly endothelium dependent and involves arachidonic acid degradation products, NO and big-conductance Ca^2+^-activated K^+^ channels (BK_Ca_) and, in addition, is related to the activation of O-1918-sensitive receptors but not cannabinoid CB_1_, CB_2_ or vanilloid TRPV1 receptors.

## Materials and methods

All protocols were approved by the Human Ethical Committee of the Medical University of Białystok (no. R-I-002/309/2008). Tissue donors had provided informed consent for the use of their vessels.

### Pulmonary artery preparation

Human lung tissue was obtained from 41 patients (35 men and 6 women, mean age of 66.4 ± 0.7 years including 88 % smokers) undergoing lobectomy or pneumonectomy during resection of lung carcinoma. Pre-operative echocardiography revealed normal left and right ventricular function in each case. Patients did not have any clinical evidence of pulmonary hypertension. Before the operation, all patients received cephalosporins and low-molecular-weight heparin as infection and thrombosis prophylaxis, respectively. The tissue was transported to the laboratory within half an hour in a cold (4 °C), pre-gassed Tyrode’s bicarbonate solution (for composition, see below at section “Organ bath technique”). Lobar and segmental hPA branches were cleaned from the lung parenchyma and cut into rings (from the middle portion of each artery, 3- to 5-mm length and 2- to 4-mm outer diameter).

### Organ bath technique

The arterial rings were suspended on stainless steel wires in 10-ml organ baths containing Tyrode’s solution (concentration in millimolar: NaCl, 139.2; KCl, 2.7; CaCl_2_, 1.8; MgCl_2_, 0.49; NaHCO_3_, 11.9; NaH_2_PO_4_, 0.4; glucose, 5.5) and were gassed continuously with 95 % O_2_ and 5 % CO_2_, at 37 °C and pH 7.4. Pulmonary artery rings were allowed to equilibrate for 90 min; during this time period, the bath fluid was exchanged every 10 min with fresh Tyrode’s solution. The optimal resting tension was about 20–25 mN (depending on the rings’ internal diameter), which ensured that responses to agonists were maximal. Muscle tension was recorded by a force displacement transducer (PIM 100RE, BIO-SYS-TECH, Białystok, Poland) and displayed on a computer.

After the equilibration period, all rings were pre-constricted sub-maximally with (–) phenylephrine (1 μM) to prime the tissues and to assess the integrity of the endothelium (at least 80 % relaxation in response to acetylcholine 1 μM was designated as endothelium-intact). When necessary, the endothelium was removed by gentle rubbing of the intima. The absence of acetylcholine-induced vasorelaxation was verified as successful endothelial denudation.

Viable vessels were constricted sub-maximally with U-46619 (0.01–0.03 μM; a prostanoid TP receptor agonist). In some experiments, serotonin (5-HT, 1 μM) or high-KCl (60 mM) Tyrode’s solution prepared by equimolar substitution of NaCl by KCl was used to pre-constrict the rings. When a stable level of tone was maintained (after about 45 min of exposure time), concentration-response curves (CRCs) were generated by cumulative application of anandamide. The agonist was added in steps of 0.5 log units with about 8–10 min between each application. Controls were obtained by the addition of Tocrisolve. All experiments were performed in paired vessels; control and anandamide-treated responses were studied on vessels from the same patient. Only one curve was determined in each individual preparation. In an additional series of experiments, complete CRCs of U-46619 were recorded 30 min after administration of anandamide 10 μM or Tocrisolve.

To determine the mechanisms of the vasorelaxant effects of anandamide, some tissues were pre-treated for 30 min (if not stated otherwise) with the following antagonists/inhibitors alone or in combination (details are provided in brackets): 30-min treatment with AM251 1 μM (cannabinoid CB_1_ receptor), SR144528 1 μM (CB_2_ receptor), O-1918 10 μM (endothelial cannabinoid receptor), RO1138452 1 μM (prostanoid IP receptor), URB597 1 μM (FAAH), 45-min incubation with capsazepine 1 μM (vanilloid TRPV1 receptor), indomethacin 10 μM (COX-1/COX-2), nimesulide 10 μM (COX-2), *N* ^*G*^-nitro-l-arginine methyl ester (l-NAME) 300 μM (NO synthase), and iberiotoxin 0.1 μM (large-conductance Ca^2+^-activated K^+^ channels). Antagonists/inhibitors were also present during the construction of the CRCs.

The resting tension was not affected by most of the drugs but slightly decreased by nimesulide and ethanol. Therefore, slightly different U-46619 concentrations (0.01–0.03 μM) were used in experiments with the latter agents to achieve comparable contraction levels (see Table 1 for details). URB597, RO1138452, capsazepine, nimesulide and O-1918 were dissolved in ethanol, and the effects of the appropriate vehicle on the relaxation to anandamide were also tested when the final bath concentration exceeded 0.1 % (*v*/*v*). No significant effects of vehicle on the CRCs were observed (see Table 1 for details).

**Table 1:** Influence of various treatments on the relaxant effect to anandamide in endothelium-intact human pulmonary arteries pre-constricted with U-46619, serotonin or KCl

Group	*n*	Tension (mN)	pEC_25_	pEC_50_	*R*_max_ (%)^a^
Control (0.01–0.03 μM U-46619 pre-constricted)	21	10.0 ± 0.4	5.7 ± 0.1	5.0 ± 0.1	90 ± 7
–Endothelium	7	13.7 ± 2.8	–	–	23 ± 11***
l-NAME (300)	4	10.0 ± 1.1	4.2 ± 0.1***	–	36 ± 10***
Indomethacin (10)	7	10.7 ± 1.8	–	–	28 ± 10***
l-NAME (300) + indomethacin (10)	3	8.4 ± 0.8	–	–	11 ± 9***
RO1138452 (1)	5	10.0 ± 2.0	4.5 ± 0.3**	–	30 ± 5***
Iberiotoxin (0.1)	5	8.7 ± 1.0	4.5 ± 0.1***	–	39 ± 8
URB597 (1)	12	12.9 ± 1.5	5.1 ± 0.2*	4.3 ± 0.1**	65 ± 3**
URB597 (1) + AM251 (1)	7	10.0 ± 1.8	5.2 ± 0.2	4.4 ± 0.1	63 ± 9
URB597 (1) + SR144528 (1)	6	10.9 ± 2.2	4.8 ± 0.2	4.7 ± 0.1	76 ± 10
URB597 (1) + capsazepine (1)	7	10.5 ± 1.2	4.8 ± 0.1	4.5 ± 0.2	72 ± 14
URB597 (1) + O-1918 (10)	7	10.3 ± 1.5	4.4 ± 0.1^†^	–	38 ± 3^†^
Control (ethanol 0.1 % *v*/*v*)	5	7.9 ± 2.0	6.0 ± 0.1	5.1 ± 0.2	97 ± 9
Nimesulide (10)	5	7.1 ± 1.0	5.2 ± 0.4*	4.5 ± 0.1*	75 ± 15
Control (1 μM serotonin pre-constricted)	5	8.8 ± 0.5	5.3 ± 0.2	4.8 ± 0.1	85 ± 8
Control (60 mM KCl pre-constricted)	5	11.4 ± 2.5	–	–	25 ± 12^°°°^

Data are expressed as the mean ± SEM of *n* independent experiments

Some experiments were performed in endothelium-denuded rings (–endothelium). If not stated otherwise, micromolar concentrations of chemicals are provided in brackets

*l* *-NAME N* ^*G*^-nitro-l-arginine methyl ester

*P < 0.05; **P < 0.01; ***P < 0.001, compared with the respective control group; ^†^ *P* < 0.05, compared with URB597 alone; ^°°°^ *P* < 0.001, compared with the control obtained in the presence of U-46619

^a^Relaxant effect at the highest anandamide concentration (100 μM)

### Preparation of tissue extracts

The hPAs and lungs were homogenized (20 % *w*/*v*) in 0.05 mol/l Tris-HCl, pH 7.6, with the use of a knife homogenizer (Polytron) and were subsequently sonicated at 0 °C. Homogenates were centrifuged at 16,000 × *g* for 30 min at 4 °C. The supernatant was used for protein determination (Bradford method) and Western blot analysis.

### Western blot analysis

Slab sodium dodecyl sulphate polyacrylamide gel electrophoresis (SDS/PAGE) was used, according to the method by Laemmli (1970). Equal amounts (about 100 μg) of protein were electrophoresed. After SDS/PAGE, the gels were allowed to equilibrate for 5 min in 25 mM Tris plus 0.2 M glycine in 20 % (*v*/*v*) methanol. The proteins were transferred to 0.2 μm pore-sized nitrocellulose at 25 mA for 1 h by using a LKB 2117 Multiphor II electrophoresis unit. The nitrocellulose was incubated with rabbit polyclonal anti-FAAH antibody at a concentration of 1:500 and rabbit polyclonal anti-β-actin antibody at a concentration of 1:500 in 5 % dried milk in Tris-buffered saline with Tween 20 (TBS-T) (20 mM Tris-HCl buffer, pH 7.4, containing 150 mM NaCl and 0.05 % Tween 20) overnight. In order to analyze FAAH and β-actin, a second antibody, i.e. anti-rabbit IgG (whole molecule) conjugated with alkaline phosphatase, was added at a concentration of 1:5000 in TBS-T and incubated for 1 h under continuous shaking. Then nitrocellulose was washed with TBS-T (5 × 10 min) and submitted to 5-bromo-4-chloro-3-indolyl phosphate/nitro blue tetrazolium (BCIP/NBT) liquid substrate reagent.

### Drugs used

Acetylcholine chloride, (–) phenylephrine hydrochloride, serotonin (creatinine sulphate complex) and l-NAME (Sigma, Munich, Germany) were dissolved in deionized water. Indomethacin (Sigma) was dissolved in 0.5 M NaHCO_3_. Anandamide was purchased from Tocris (Bristol, UK) as a 10-mg/ml emulsion in soya water (1:4) and was further diluted with deionized water. Stock solutions (10 mM) of capsazepine, AM251 (*N*-(piperidin-1-yl)-5-(4-iodophenyl)-1-(2,4-dichlorophenyl)-4-methyl-1*H*-pyrazole-3-carboxamide), nimesulide (Tocris), URB597 (3′-(aminocarbonyl)[1,1′-biphenyl]-3-yl)-cyclohexylcarbamate), RO1138452 (4,5-dihydro-*N*-[4-[[4-(1-methylethoxy)phenyl]methyl]phenyl]-1*H*-imidazol-2-amine) (Cayman Chemicals, Ann Arbor, MI, USA) and SR 144528 (*N*-[(1S)-endo-1,3,3-trimethyl bicyclo[2.2.1]heptan-2-yl]-5-(4-chloro-3-methylphenyl)-1-(4-methylbenzyl)-pyrazole-3-carboxamide) (Sanofi-Aventis, Montpellier, France) were prepared in ethanol and further diluted in distilled water. U-46619 ((5Z)-7-[(1R,4S,5S,6R)-6-[(1E,3S)-3-hydroxy-1-octenyl]-2-oxabicyclo[2.2.1]hept-5-yl]-5-heptenoic acid) and O-1918 (1,3-dimethoxy-5-methyl-2-[(1R,6R)-3-methyl-6-(1-methylethenyl)-2-cyclohexen-1-yl]-benzene) (Cayman) were supplied in methyl acetate, which was evaporated under a stream of nitrogen, and ethanol was added as a solvent. These agents were diluted to their final concentrations with deionized water. Anti-FAAH antibodies were purchased from Cayman (cat. no. 101600) and anti-β-actin and anti-rabbit IgG alkaline phosphatase antibodies from Sigma-Aldrich (St. Louis, MO, USA).

### Calculations and statistical analysis

Contractile responses to U-46619 are shown as a percentage of the contraction induced by 60 mM KCl (Kozłowska et al. 2007, 2008). Vasodilatory effects of anandamide or its solvent are expressed as the percentage relaxation of the isometric contraction induced by U-46619, 5-HT or KCl. In analogy to our previous study (Baranowska-Kuczko et al. 2012), the extent of relaxation at 100 μM anandamide was quantified as a rough measure of the maximum extent of relaxation obtainable (*R*_max_). Since some experimental procedures attenuated the *R*_max_ of anandamide by more than 50 %, the concentrations causing a relaxation of 25 % of the pre-contracted vessel were given (effective concentration for 25 % (EC_25_) values). The half maximal effective concentration (EC_50_) values (i.e. the concentrations causing a relaxation of 50 % of the pre-contracted vessel) were used when *R*_max_ was at least 60 %. These values were calculated from the individual CRCs and are usually expressed as their negative decadic logarithms (pEC_25_ and pEC_50_).

The rightward shift of the CRC of anandamide by the different experimental procedures (endothelium removal, enzyme blockers and receptor antagonists) was determined on the basis of the EC_25_ values. The results are expressed as the mean ± SEM of *n* experiments; *n* always refers to the number of patients. For statistical analysis, Student’s *t* test for unpaired data was used. For comparisons of the CRCs of two or more treatment groups to the same control, analysis of variance (ANOVA) followed by Dunnett’s test was performed (Prism 5, GraphPad Software, Inc., La Jolla, CA, USA). Differences were considered significant when *P* < 0.05.

## Results

### Influence of anandamide on the hPAs pre-constricted with U-46619 or serotonin

**Fig. 1:**
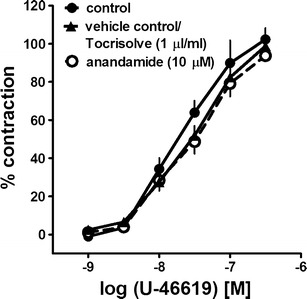
Influence of anandamide and its solvent Tocrisolve on the concentration-dependent vasoconstriction induced by U-46619 in the endothelium-intact human pulmonary artery. The results are expressed as percentage of the isometric contraction induced by KCl 60 mM. Note that anandamide (although relaxing pre-constricted vessels) did not affect the basal tone. The mean ± SEM of three to four tissues for each curve is presented. The SEM is smaller than or equal to the size of symbols in few cases

The prostanoid TP receptor agonist U-46619 (0.001–0.3 μM) induced a concentration-dependent contraction of hPA rings (pEC_50_ = 7.7 ± 0.1, *n* = 3; Fig. 1). Anandamide 10 μM and its vehicle Tocrisolve (1 μl/ml) did not modify the CRC of U-46619 (pEC_50_ = 7.5 ± 0.1, *n* = 3; 7.5 ± 0.1, *n* = 4; Fig. 1) nor did they affect the basal tone of hPA rings. In most experiments, U-46619 was used to pre-contract the hPAs. The tension generated by U-46619 at 0.01–0.03 μM (concentrations approximately equivalent to its EC_60_) did not differ significantly from that induced by 5-HT 1 μM (approximately equivalent to its EC_60_; Kozłowska et al. 2007, 2008) or KCl 60 mM (Table 1).

**Fig. 2:**
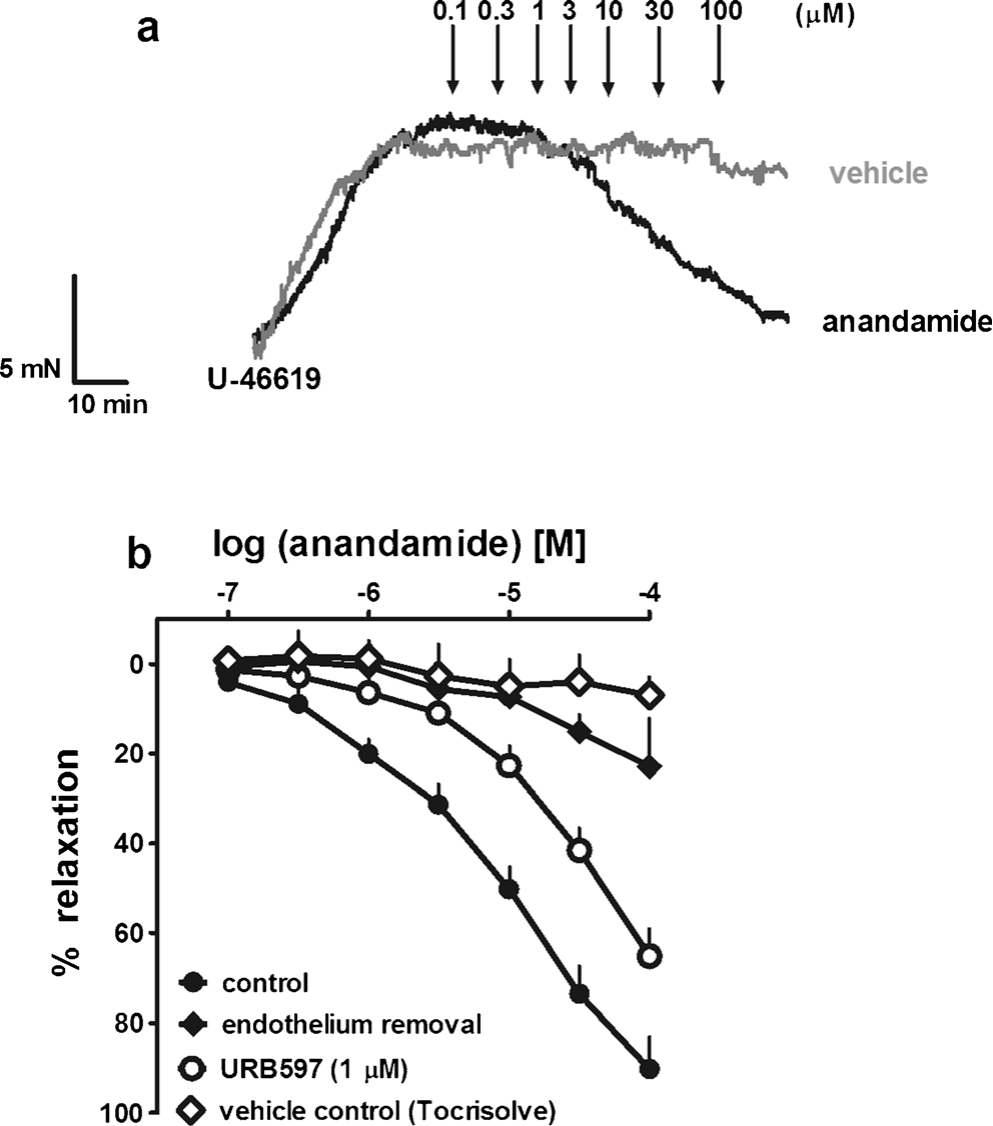
Representative original traces of the effects of anandamide and its vehicle (Tocrisolve) (**a**) and influence of endothelium removal and URB597 on the relaxant effect of anandamide in the human pulmonary artery (**b**). **a** The experiments were performed in separate vessels obtained from the same patient. The *arrows* indicate the application of the particular concentrations of anandamide or of its vehicle. **b** The results are expressed as the percentage relaxation of the isometric contraction induced by U-46619. The effects of Tocrisolve (0.001–1.0 % *v*/*v*; vehicle of anandamide) are shown as well. The mean ± SEM of 3–21 tissues for each curve is presented. The SEM is smaller than or equal to the size of the symbols in few cases

**Fig. 3:**
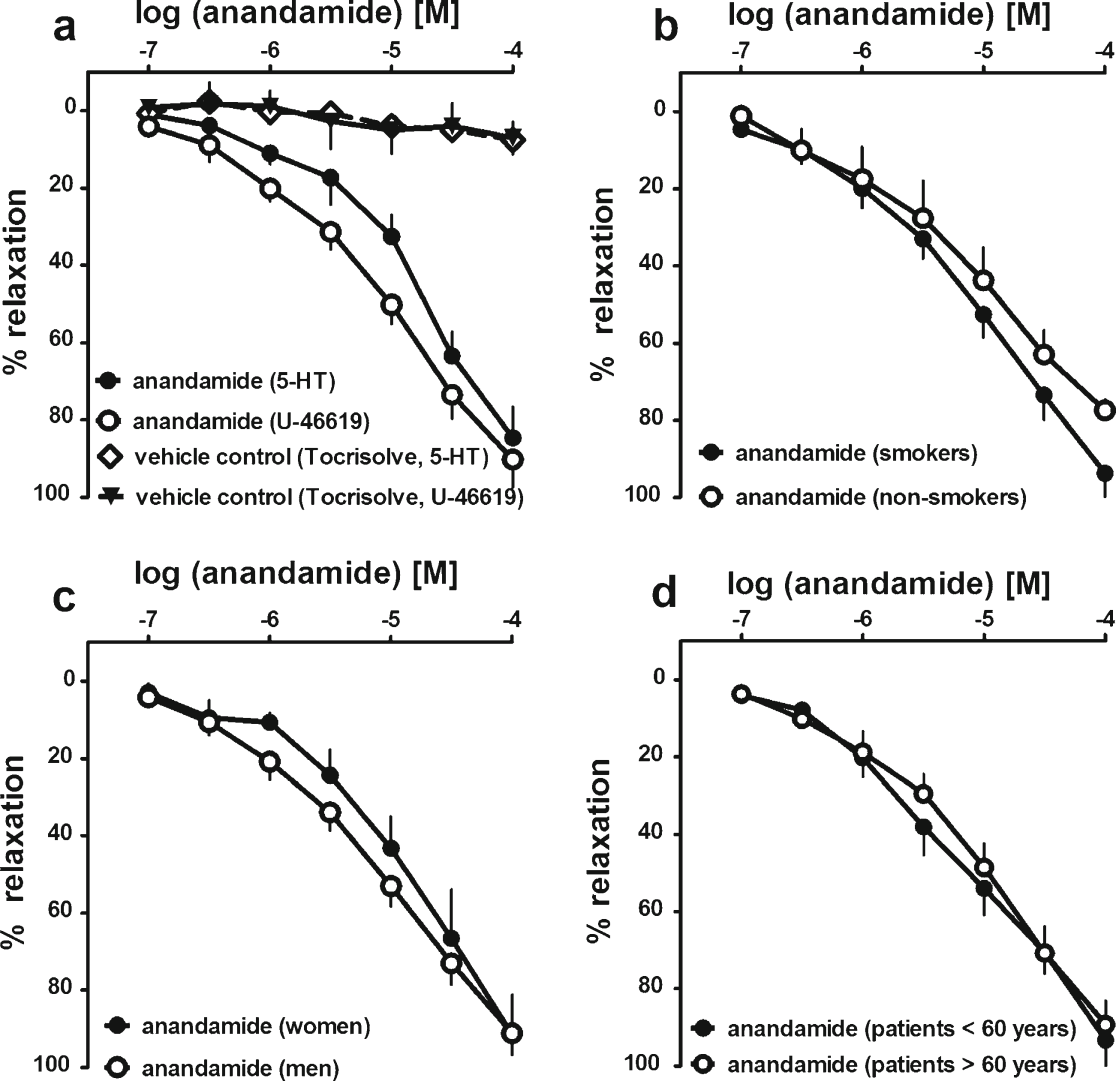
Influence of the pre-constricting agent (**a**), smoking habits (**b**), sex (**c**) and age (**d**) of the patients on the relaxant effect of anandamide in the endothelium-intact human pulmonary artery. The results are expressed as the percentage relaxation of the isometric contraction induced by serotonin (5-HT) (**a**) or U-46619 (**a**–**d**). Results are given as the mean ± SEM of the number of tissues (patients), namely **a** *n*=5 5-HT-pre-constricted and *n*=21 U-46619-pre-constricted, **b** 17 smokers and 4 non-smokers, **c** 6 women and 15 men and **d** 6 patients <60 years (54.4 ± 1.2) and 15 patients >60 years (67.1 ± 0.7; *P* < 0.001). Curves did not differ with respect to the *R*_max_ (vasorelaxant effect of anandamide 100 μM) and the pEC_50_ (range of 4.8–5.1). The SEM is smaller than or equal to the size of symbols in few cases

Anandamide caused almost full relaxation of the hPAs pre-constricted with U-46619 (Fig. 2); the pEC_25_ and pEC_50_ values were 5.7 and 5.0, respectively (Table 1). The vasodilatation induced by anandamide was gradual in onset. Thus, it took 80 min to construct the whole CRCs. A typical trace of anandamide-induced relaxations in U-46619-pre-constricted arteries is shown in Fig. 2a. The following parameters had virtually no influence on the anandamide-evoked relaxation: use of 5-HT instead of U-46619 as pre-contracting agent (Fig. 3a), smoking habits (Fig. 3b), sex (Fig. 3c) and age (Fig. 3d).

### Effects of endothelium removal and URB597 on the relaxation to anandamide

Removal of the endothelium very strongly reduced the vasodilator effect of anandamide (Fig. 2b, Table 1). Pre-treatment with the FAAH inhibitor URB597 (1 μM) attenuated the anandamide-induced vasorelaxation in endothelium-intact hPAs (Fig. 2b), yielding a rightward shift by a factor of 4. Moreover, the effect elicited by the highest concentration of the agonist was reduced by about 25 % (for pEC_25_ and *R*_max_ values, see Table 1).

### Effects of l-NAME, COX inhibitors and RO1138452 on the relaxation to anandamide

**Fig. 4:**
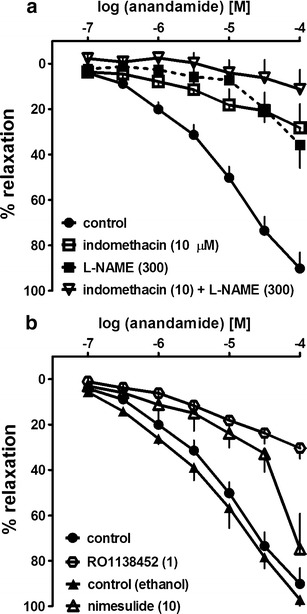
Influence of **a** indomethacin, *N* ^*G*^-nitro-l-arginine methyl ester (l-NAME) given separately or in combination, and **b** nimesulide and RO1138452 on the relaxant effect of anandamide in the endothelium-intact human pulmonary artery. The results are expressed as the percentage relaxation of the isometric contraction induced by U-46619. The control concentration-response curves were obtained in the absence (related to indomethacin, l-NAME and RO1138452) or presence of ethanol (related to nimesulide). The mean ± SEM of 3–21 tissues for each curve is presented. The SEM is smaller than or equal to the size of symbols in few cases

As shown in Fig. 4a, the NO synthase inhibitor l-NAME (300 μM) and the COX-1/COX-2 inhibitor indomethacin (10 μM) given separately or in combination reduced by about 55, 60 or 80 %, respectively, the relaxant effect elicited by the highest concentration of anandamide (100 μM) (for *R*_max_ value, see Table 1). Moreover, l-NAME shifted to the right of the CRC for anandamide by a factor of 34 (for pEC_25_ value, see Table 1). The selective COX-2 inhibitor nimesulide (10 μM) shifted to the right of the CRC for anandamide by a factor of 6; the effect elicited by the highest anandamide concentration was reduced by about 20 % (Fig. 4b; for pEC_25_, pEC_50_ and *R*_max_ values, see Table 1). The prostanoid IP receptor antagonist RO1138452 (1 μM) shifted the CRC for anandamide to the right by a factor of 16 and reduced the maximal effect of anandamide at 100 μM by about 60 % (Fig. 4b; for pEC_25_ and *R*_max_ values, see Table 1).

### Effects of KCl and the K^+^ channel blocker iberiotoxin on the relaxation to anandamide

**Fig. 5:**
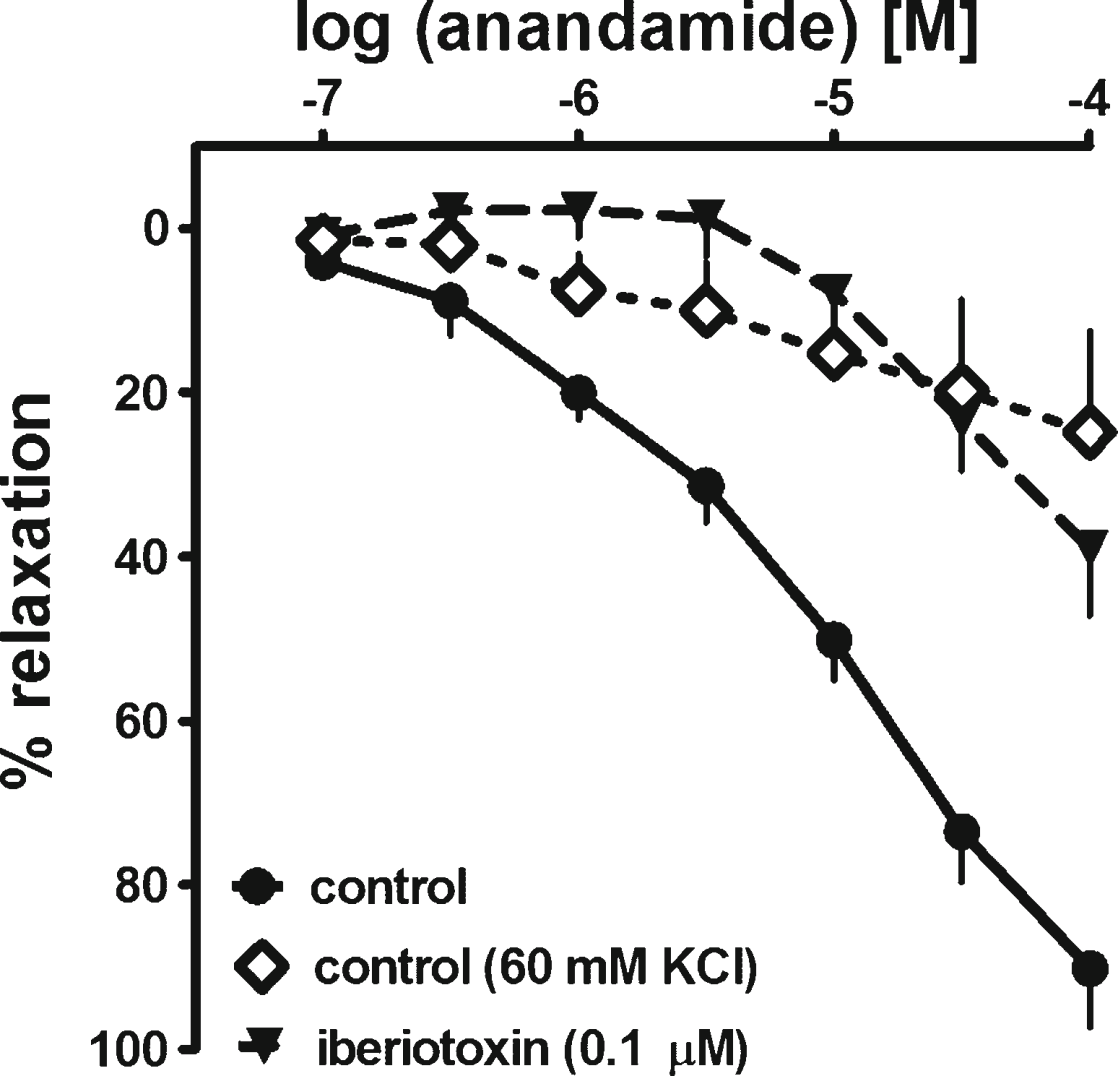
Influence of KCl and iberiotoxin on the anandamide-induced relaxation in the endothelium-intact human pulmonary artery. The results are expressed as the percentage relaxation of the isometric contraction induced by KCl (*diamonds*) and U-46619 (*circles* and *inverted triangles*). The mean ± SEM of 5–21 tissues for each curve is presented. The SEM is smaller than or equal to the size of symbols in few cases

In order to determine if potassium channels contribute to the vasodilator effects of anandamide, some experiments were carried out in KCl pre-constricted arteries and compared with U-46619-pre-constricted vessels. The vasorelaxation to anandamide at 100 μM was reduced by about 65 % in KCl pre-constricted arteries compared with arteries pre-constricted with U-46619 (Fig. 5; for *R*_max_ value, see Table 1). Pre-treatment with the selective blocker of big-conductance Ca^2+^-activated potassium channels, iberiotoxin (0.1 μM), produced a 16-fold rightward shift of the CRC for anandamide in U-46619-pre-constricted arteries; the effect of anandamide 100 μM was reduced by 50 % (Fig. 5; pEC_25_ and *R*_max_ values in Table 1).

### Effects of cannabinoid and TRPV1 receptor antagonists on the relaxation to anandamide

**Fig. 6:**
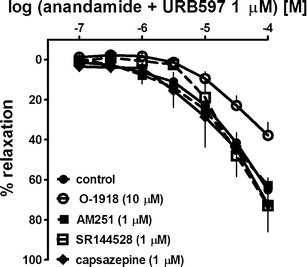
Influence of AM251, SR144528, capsazepine and O-1918 on the relaxant effect of anandamide in the presence of URB597 (1 μM) in the endothelium-intact human pulmonary artery. The results are expressed as the percentage relaxation of the isometric contraction induced by U-46619. The mean ± SEM of 6–12 tissues for each curve is presented. The SEM is smaller than or equal to the size of symbols in few cases

As described above, the FAAH inhibitor URB597 (1 μM) attenuated the anandamide-induced relaxation in hPAs pre-constricted with U-46619. Therefore, the potential involvement of cannabinoid and vanilloid receptors in the vasodilatory response to anandamide was examined in endothelium-intact hPAs treated with URB597. The CB_1_ receptor antagonist AM251 (1 μM), the CB_2_ receptor antagonist SR144528 (1 μM) and the TRPV1 receptor antagonist capsazepine (1 μM) did not affect the anandamide-induced relaxation (Fig. 6). On the other hand, O-1918 (10 μM), an antagonist of the putative endothelial cannabinoid receptor, produced a fivefold rightward shift of the CRC for anandamide and reduced the relaxant effect of the highest concentration of anandamide (100 μM) by about 25 % (Fig. 6; for pEC_25_ and *R*_max_ values, see Table 1).

### Expression of FAAH in the human pulmonary artery and lung

**Fig. 7:**
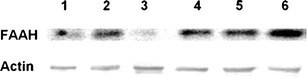
Western blots for fatty acid amide hydrolase (FAAH) protein in endothelium-intact human pulmonary arteries (lanes 1–3) and lungs (lanes 4–6). Samples used for electrophoresis consisted of 100 μg of protein of pooled tissue extracts (*n* = 6) obtained from three different experiments. The selectivity of the FAAH antibody was proved previously by Fowler et al. (2001). β-actin (43 kDa) was used as a loading control

The expression of FAAH protein in the endothelium-intact pulmonary artery (Fig. 7, lanes 1–3) and in the lung of humans (Fig. 7, lanes 4–6) was analyzed by Western blotting with polyclonal antibodies against FAAH. Western blot analysis showed a single immunoreactive band of the molecular size expected for FAAH (63 kDa) (Fig. 7).

## Discussion

The aim of the present study was to examine the receptor(s) and mechanism(s) involved in the vasodilatory effect of AEA in hPAs. The thromboxane analogue U-46619 was used as a vasoconstrictor agent to facilitate comparisons with our work on rat pulmonary arteries (Baranowska-Kuczko et al. 2012). Moreover, thromboxane is important for the maintenance of the pulmonary tone and implicated in the development of PAH (Anderson and Nawarskas 2010).

In the present study, anandamide caused a slowly developing relaxation of the endothelium-intact hPAs yielding a *R*_max_ of ~90 % and a pEC_50_ of ~5.0. The possibility that the relaxant effect of anandamide is related to a direct antagonistic effect of anandamide at prostanoid TP receptors in hPAs could be excluded since anandamide at a concentration that evoked ~50 % relaxation (10 μM) did not influence the CRC of U-46619. The fact that anandamide showed similar potencies in preparations pre-contracted with U-46619 and serotonin (pEC_50_ values of 5.0 and 4.8, respectively) also argues against this possibility. A similar potency of anandamide was also obtained in pulmonary arteries of the rat (pEC_50_ 5.0; Baranowska-Kuczko et al. 2012). Moreover, the potency of anandamide in the hPAs resembled that of virodhamine (pEC_50_ 5.1; Kozłowska et al. 2008). As in our previous studies, the concentrations of anandamide were in the micromolar range and similar to those occurring under pathophysiological conditions (Malinowska et al. 2012) when endocannabinoid levels are enhanced and may be relevant for vascular performance in pulmonary disorders.

The majority (~90 %) of hPAs used in our study was obtained from smokers. This might mean that the effect of anandamide represents an abnormal rather than the real physiological response. This possibility, however, is very unlikely since there was no difference in the response to this agonist in tissue taken from smoking and non-smoking patients. Moreover, the age and the sex of the patients do not influence the anandamide-induced vasorelaxation.

Endothelium plays an important physiological role in the regulation of smooth muscle tone in the pulmonary circulation. We found that the relaxant effect of anandamide on the hPAs is to a large extent endothelium-dependent. Thus, all further experiments were carried out in rings with intact endothelium. The NO synthase inhibitor l-NAME (300 μM; Kozłowska et al. 2008) strongly inhibited the anandamide-induced relaxation.

Anandamide is metabolized into arachidonic acid and ethanolamine by FAAH. Indeed, FAAH metabolism products are involved in the anandamide-induced relaxation in hPAs. First, we showed the FAAH expression in human lungs and pulmonary arteries (and could confirm the results obtained by Meng et al. (2008) and Wenzel et al. (2013) on hPA smooth muscle cells). Second, the FAAH inhibitor URB597 (1 μM; Baranowska-Kuczko et al. 2012) reduced the anandamide-evoked relaxation in the hPAs. The anandamide-derived arachidonic acid may be converted via COX-1 and/or COX-2 to vasoactive eicosanoids, yielding prostaglandins and prostacyclin. Both the non-selective COX inhibitor indomethacin (10 μM; Baranowska-Kuczko et al. 2012) and the selective COX-2 antagonist nimesulide (10 μM; Wahn et al. 2005) diminished the anandamide-induced relaxation. The reason why the attenuation of the vasorelaxation by selective COX-2 inhibition was less marked when compared with combined COX-1 plus COX-2 inhibition may be that an exposure to anandamide lasting for >1 h is required to fully induce COX-2 expression (Chen et al. 2005).

In the pulmonary circulation, the IP receptor is detected (Li et al. 2012) and prostacyclin is a particularly important product of COX (Kuwano et al. 2008; Waxman and Zamanian 2013; Benyahia et al. 2013). We found that the IP receptor antagonist RO1138452 (1 μM; Kozłowska et al. 2012; Baranowska-Kuczko et al. 2012) reduced the anandamide-mediated relaxation of the hPAs. Moreover, the extent of attenuation of the anandamide-evoked relaxation is similar to that obtained with indomethacin. Our data suggest that the IP receptor is involved in the anandamide-mediated response in the hPAs and are comparable to the results obtained in the rat pulmonary arteries (pA_2_ = 6.2; Baranowska-Kuczko et al. 2012). Since the effect of l-NAME and indomethacin almost equalled that of endothelial removal and the dual inhibition of NO and COX-1/2 and almost completely abolish the effect of anandamide, one can conclude that the endothelium-dependent relaxation of the hPAs induced by anandamide is mainly due to NO and COX-dependent pathways.

Two sets of experiments suggest the involvement of potassium channels in the effect of anandamide. First, the relaxation to anandamide was almost completely abolished in the hPAs pre-constricted with a high concentration of KCl (60 mM; Kozłowska et al. 2008). Such experimental conditions depolarize the hPAs by inhibiting the potassium ion efflux. Second, iberiotoxin (0.1 μM; Galvez et al. 1990), a selective inhibitor of the large-conductance calcium-dependent potassium channels, strongly reduced the relaxant effect of anandamide. The BK_Ca_ channels may be activated by vasoactive anandamide degradation products (arachidonic acid and derivatives; Gauthier et al. 2005) or by NO in the hPAs (Guerard et al. 2004). In addition, increased intracellular Ca^2+^ could release endothelial vasorelaxants, including NO and PGI_2_ (Félétou 2009). It is therefore possible that COX-dependent products of AEA-derived arachidonic acid induce the release of NO which in turn acts in the hPA via K_Ca_ channel opening.

Anandamide mediates its cardiovascular effects through cannabinoid CB_1_ and CB_2_ receptors, the O-1918-sensitive endothelial cannabinoid receptor and the vanilloid TRPV1 receptor (Montecucco and Di Marzo 2012; Malinowska et al. 2012). To eliminate the influence of anandamide metabolites, all experiments aimed at the determination of cannabinoid-sensitive receptors were performed in the presence of URB 597 (1 μM). CB_1_ receptors are not involved in the AEA-mediated vasorelaxation since the respective receptor antagonist AM251 (1 μM; Wheal et al. 2010) did not influence the CRC of anandamide. The involvement of CB_2_ and vanilloid TRPV1 receptors could also be excluded since the respective antagonists at these receptors, SR144528 (1 μM; Kozłowska et al. 2008) and capsazepine (1 μM; Tamaki et al. 2012), failed to modify the vasorelaxant effect of anandamide. By contrast, O-1918 (10 μM; pA_2_ = 6.3 and 6.0; Kozłowska et al. 2008; Baranowska-Kuczko et al. 2012, respectively) shifted to the right of the CRC for anandamide, suggesting that this receptor plays a role in the anandamide-induced vasorelaxation.

The quantitative role of unmetabolized anandamide appears to be lower than that played by the COX product. The mechanism behind the vasodilator effect of unmetabolized anandamide has not been studied in the present work; however, in our previous paper on the same preparation, abnormal cannabidiol (which like anandamide activates the endothelial cannabinoid receptor but is not metabolized) was shown to act via the endothelium and the activation of Ca^2+^-activated potassium channels (Kozłowska et al. 2007).

The present work (in combination with our previous studies: Kozłowska et al. 2007; 2008) on hPA is qualitatively similar to our study on the rat pulmonary artery (Baranowska-Kuczko et al. 2012); the extent of vasodilatation was even more marked in the human than in the rat pulmonary artery. In those experiments, pre-constricted pulmonary arteries were used; in non-pre-constricted vessels, anandamide had no effect at all in pulmonary arteries of rats (Baranowska-Kuczko et al. 2012), humans (present study) and mice (Wenzel et al. 2013). In the isolated perfused lung of the mouse (Wenzel et al. 2013) and the rabbit (Wahn et al. 2005), anandamide caused an increase in the perfusion pressure (reflecting a vasopressor response). The isolated lung model mainly reflects vessels of the pulmonary circulation with a small calibre. Although the data obtained from the isolated perfused lung are of particular interest with respect to in vivo conditions, one has to consider that, in the pulmonary unlike the systemic circulation, there are no typical resistance vessels (Saouti et al. 2010). In order to have a comprehensive view, it therefore appears necessary to consider the contradictory results obtained on the pulmonary arteries and in the isolated perfused lung preparation in combination. The reason why anandamide acts so differently in the two experimental models is unclear but it is of interest that this endocannabinoid behaved as a vasodilator, vasoconstrictor or was without vascular activity also in vessels of the systemic circulation, depending on the calibre and/or the vascular tone (White and Hiley 1998; Vanheel and Van de Voorde 2001). With respect to the results in the pulmonary circulation, it is intriguing that the pathways for vasodilatation and vasoconstriction resemble each other. So, FAAH plays a role both for endothelium-dependent vasodilatation (Baranowska-Kuczko et al. 2012; present study) and endothelium-independent vasoconstriction (Wahn et al. 2005; Wenzel et al. 2013). With respect to the subsequent step, COX is implicated both in the vasodilatation (Baranowska-Kuczko et al. 2012; present study) and vasoconstriction (Wahn et al. 2005; Wenzel et al. 2013). Thus, one should be somewhat more cautious when suggesting the potential use of FAAH inhibitors for the treatment of PAH (Wenzel et al. 2013) or other diseases (e.g. Bisogno and Maccarrone 2013).

## Conclusions

In conclusion, our findings show that anandamide relaxes hPAs. The vascular response to anandamide is almost totally endothelium-dependent and involves the opening of potassium channels and stimulation of NO release that, in turn, may be the result of activation of the putative O-1918-sensitive cannabinoid receptor or production of COX-sensitive prostacyclin-like vasoactive products.
